# Allopregnanolone: An overview on its synthesis and effects

**DOI:** 10.1111/jne.12996

**Published:** 2021-06-29

**Authors:** Silvia Diviccaro, Lucia Cioffi, Eva Falvo, Silvia Giatti, Roberto Cosimo Melcangi

**Affiliations:** ^1^ Dipartimento di Scienze Farmacologiche e Biomolecolari Università degli Studi di Milano Milano Italy

**Keywords:** neuroactive steroids, neurodegenerative disorders, psychiatric disorders, progesterone metabolism, sex difference

## Abstract

Allopregnanolone, a 3α,5α‐progesterone metabolite, acts as a potent allosteric modulator of the γ‐aminobutyric acid type A receptor. In the present review, the synthesis of this neuroactive steroid occurring in the nervous system is discussed with respect to physiological and pathological conditions. In addition, its physiological and neuroprotective effects are also reported. Interestingly, the levels of this neuroactive steroid, as well as its effects, are sex‐dimorphic, suggesting a possible gender medicine based on this neuroactive steroid for neurological disorders. However, allopregnanolone presents low bioavailability and extensive hepatic metabolism, limiting its use as a drug. Therefore, synthetic analogues or a different therapeutic strategy able to increase allopregnanolone levels have been proposed to overcome any pharmacokinetic issues.

## 1 INTRODUCTION

Progesterone (PROG) not only comprises a physiological regulator of reproduction,^1^, ^2^, ^3^, ^4^, ^5^ but also exerts important effects in the nervous system. Indeed, this neuroactive steroid regulates development of neurones^6^, ^7^, ^8^, ^9^ and glial cells,^10^, ^11^, ^12^, ^13^ as well as the myelination process.^14^, ^15^, ^16^, ^17^, ^18^ In addition, PROG exerts important protective effects in neurodegenerative and psychiatric disorders.^15^, ^19^, ^20^, ^21^, ^22^, ^23^, ^24^, ^25^, ^26^, ^27^ However, whether the effects of PROG are the result of itself and/or its metabolites is still poorly considered. Among PROG metabolites, the effects of allopregnanolone (ALLO), also known as tetrahydroprogesterone, in the nervous system have attracted the attention of several researchers. Therefore, even if many aspects of this neurosteroid remain to be clarified, an extensive literature on it is now available. In the present review, we discuss the state of art of this neuroactive steroid, considering its synthesis, mechanism of actions, and physiological and protective effects. In addition, whether neurodegenerative and psychiatric disorders, as well as peripheral steroid contents, influence the amount of this neuroactive steroid in the nervous system and whether sex dimorphism may occur are also taken into consideration.

## 2 SYNTHESIS AND MECHANISM OF ACTION

In the nervous system, PROG is actively converted by the enzyme 5α‐reductase (5α‐R) into dihydroprogesterone (DHP) and subsequently by the action of the enzymes 3α‐hydroxysteroid oxidoreductase or 3β‐hydroxysteroid oxidoreductase into ALLO and isoallopregnanolone (ie, the 3β‐isomer of ALLO).^28^, ^29^ Two isoforms of 5α‐R, called type 1 and type 2, are responsible for the metabolism of neuroactive steroids, including PROG.^30^, ^31^, ^32^, ^33^ Type 1 isoform is expressed in cortical, hippocampal and olfactory bulb glutamatergic neurones and in some output neurones of the amygdala and thalamus,^34^ with high levels in midbrain, corpus callosum, anterior commissure, optic chiasm, pons and spinal cord,^33^, ^35^, ^36^ and particularly in purified myelin preparations obtained from the rat brain.^35^, ^37^, ^38^ At the cellular level, this isoform has been detected in oligodendrocytes and neurones,^39^, ^40^, ^41^ in microglia^42^ and astrocytes,^39^, ^40^ and in Schwann cells.^43^, ^44^, ^45^, ^46^ Type 2 isoform is widely expressed from the forebrain to the brain stem and cerebellum of the adult rat^47^ and also highly expressed in the spinal cord, particularly in oligodendrocytes.^36^

Four human 3α‐hydroxysteroid oxidoreductase (HSOR) isozymes, but only one isoform in rats, have been cloned so far.^48^ 3α‐HSOR and 3β‐HSOR has been identified in the central nervous system (CNS)^49^; in particular, 3α‐HSOR has been detected in the rat cerebral cortex, cerebellum ^50^ and spinal cord,^36^ whereas, in the mouse brain, it is co‐localised with 5α‐R type 1 in neurones of the cerebral cortex, hippocampus, olfactory bulb, amygdala and thalamus.^34^ At the cellular level, in addition to neurones, 3α‐HSOR also appears to be highly localised in cultures of type 1 astrocytes^39^, ^40^ and oligodendrocytes.^36^, ^51^ Interestingly, the formation of ALLO by 3α‐HSOR decreases with the differentiation of oligodendrocytes.^51^

Interestingly, in the context of the growing literature regarding the role of the gut microbiota‐brain axis in human health and disease,^52^, ^53^, ^54^, ^55^, ^56^, ^57^ it is important to highlight that, as recently demonstrated, local steroidogenesis also occurs in the adult male rat colon.^58^ In particular, the levels of ALLO detected in this tissue are significantly higher than those present in plasma. In addition, the mRNA levels of 3α‐HSOR present in the adult male rat colon are significantly higher than those present in the cerebral cortex.^58^

**FIGURE 1:**
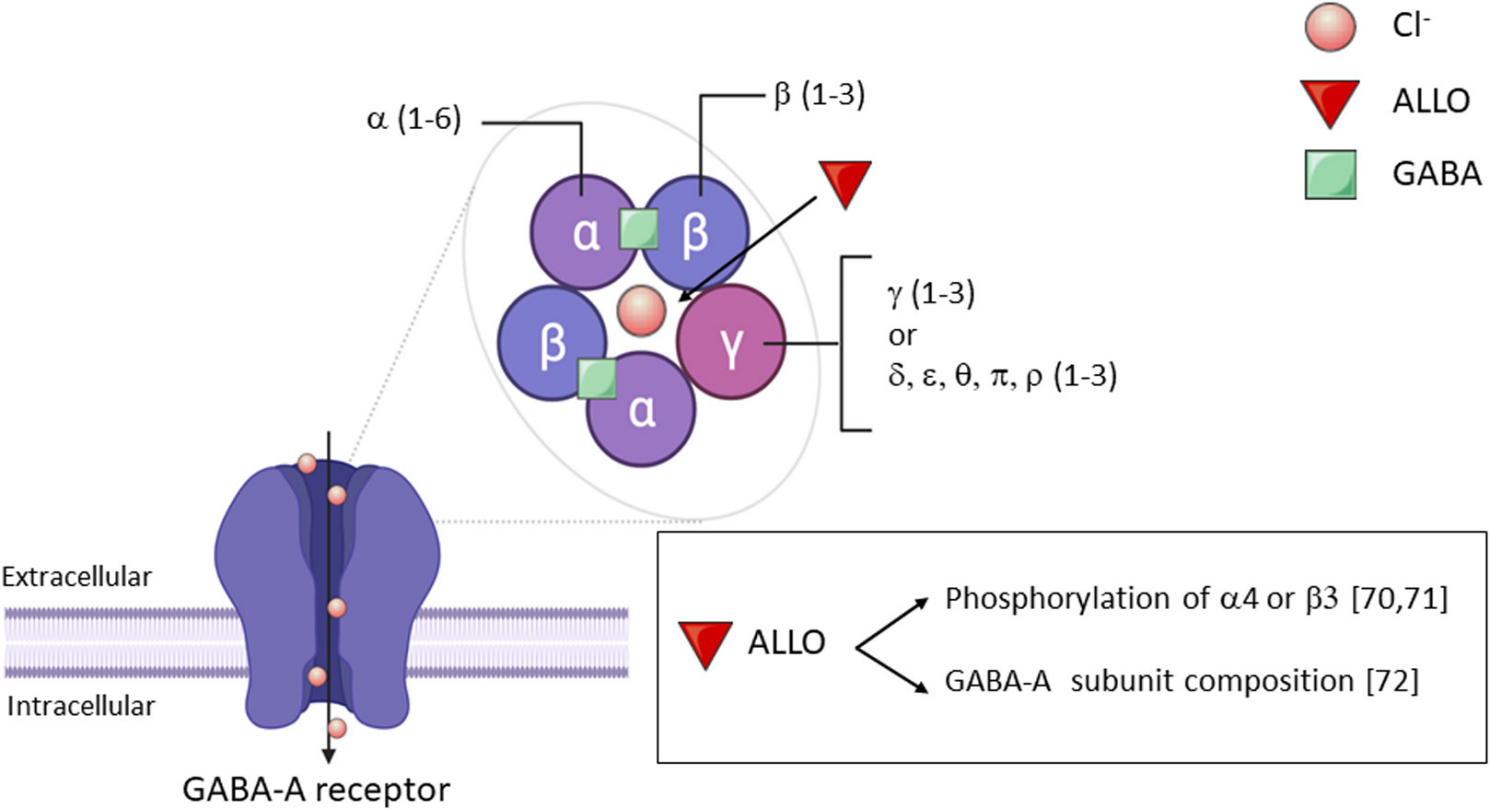
GABA_A_ receptor structure and allopregnanolone mechanism of action. The 19 different subunits of the receptor and the mechanism of action of allopregnanolone are shown. In the box: effects of allopregnanolone on GABA_A_ receptor subunit composition and phosphorylation are shown. For details, see text. ALLO, allopregnanolone; Cl‐, chloride

The metabolic conversions by the enzymes 5α‐R, 3α‐HSOR and 3β‐HSOR have a deep impact on the mechanism of action of PROG. Indeed, although DHP, similar to its precursor, is still able to interact with intracellular PROG receptor, ALLO and isoallopregnanolone interact with GABA_A_ receptor. In particular, ALLO is a potent ligand of this non‐classical steroid receptor,^59^, ^60^ whereas isoallopregnanolone does not bind directly to the GABA_A_ receptor^61^ but, instead, antagonises the effect of ALLO on the GABA_A_ receptor.^62^, ^63^ In this context, it is important to recall the molecular composition of the GABA_A_ receptor (Figure 1). This pentameric ionotropic receptor is able to respond differently to benzodiazepines, ALLO or to other modulators depending on the subunit composition. In mammals, it can consist of 19 subunits, grouped in eight classes: α(1‐6), β(1‐3), γ(1‐3), δ, ε, θ, π and ρ(1‐3).^64^ In the brain, the most common subunit combination includes two α1, two β2 and one γ2 subunits,^64^, ^65^ with a binding site for modulators placed at the interface between α and β subunits.^66^ Despite the fact that receptors containing the δ subunit, mainly located extrasynaptically, are the most sensitive to neurosteroid modulation,^67^, ^68^, ^69^ these molecules, and ALLO in particular, may affect GABA_A_ receptor function in other ways. For example, they can promote the phosphorylation of α4 or β3 subunits.^70^, ^71^ On the other hand, the composition of GABA_A_ subunits may be altered by continuous administration of PROG or ALLO^72^ (Figure 1). A deeper presentation of GABA_A_ receptor composition and ligand binding is provided in other recent reviews.^72^, ^73^, ^74^

## 3 LEVELS OF ALLO UNDER PHYSIOLOGICAL AND PATHOLOGICAL CONDITIONS

### 3.1 Physiological conditions

The first characteristic of ALLO levels is that they may differ in relation to the compartment analysed. This is a consequence of metabolism by 5α‐R and 3α‐HSOR, which is differentially expressed in the nervous system. Thus, ALLO levels show differences between the nervous system, plasma and cerebrospinal fluid (CSF), as well as between the CNS and peripheral nervous system (PNS). Moreover, they also differ between males and females on dioestrus day.^75^ In addition, the levels of PROG metabolites, such as ALLO, its precursor DHP and isoallopregnanolone, are higher in the brain of pseudopregnant females than in the male brain.^76^

Sex differences in the levels of these PROG metabolites may be the result of a sex dimorphism of the steroidogenic enzymes synthesising these molecules. Indeed, in green anole lizards, 5α‐R type 2 is higher in the brain of females than in the male brain.^77^ In rat cerebellum, 5α‐R is significantly higher in males, whereas 3α‐HSOR is significantly higher in pro‐oestrus females than in males.^50^

As observed in gonadectomised animals, the levels of ALLO in the nervous system are also influenced by its circulating levels. Interestingly, this effect shows specific features in different regions of the nervous system, being different in the two sexes and dependent on the duration of gonadal hormone deprivation.^78^ For example, in both the male cerebral cortex and cerebellum, levels of ALLO are decreased after long‐term gonadectomy (ie, 4 months), whereas these effects do not occur in the corresponding structures of the female brain.^78^ Interestingly, as reported recently, 3α‐HSOR expression in the cerebellum is also sex‐dimorphic.^50^

This neuroactive steroid is also important during brain development for adolescent and adult behaviour and for nervous system maturation.^79^ Indeed, the levels of ALLO in the forebrain of embryonic rats vary widely throughout development. During the last pregnancy period, ALLO levels sharply increase and decline prior to parturition.^80^ Some of these effects are related to a different functioning of the dorsal hippocampus, probably related to alterations in the expression of GABA receptors containing α4 and δ subunits, which are molecular alterations that can persist into adult age and can, in part, explain the reported behavioural disturbances.^81^

The levels of ALLO in the nervous system, as well as of the other PROG metabolites, are also affected by neurodegenerative and psychiatric disorders. These changes have been demonstrated to be different in males and females, in agreement with many neurodegenerative and psychiatric disorders showing sex‐dimorphic features. Some examples of them are discussed in the following subsections.

### 3.2 Pathological conditions

#### 3.2.1 Mood disorders

Several clinical and experimental observations have clearly shown that the plasma and/or CSF levels of ALLO are altered in stress‐related disorders and psychiatric diseases, such as anxiety‐like behaviour and depression, post‐partum depression and post‐partum anxiety.^82^, ^83^, ^84^, ^85^, ^86^, ^87^, ^88^, ^89^, ^90^, ^91^, ^92^ A decrease in the plasma levels of ALLO has been also observed in association with increased depression and anxiety as well as symptoms in anorexic and overweight/obese women.^93^ Interestingly, a decrease in the expression of 5α‐R type 1 enzyme has been reported in prefrontal cortex Brodmann's area 9 of depressed patients.^94^

The plasma levels of ALLO are also decreased in human alcoholics,^95^ and are altered after ethanol withdrawal in the mouse cerebral cortex and hippocampus.^96^ In agreement, polymorphic variations in the 3α‐HSOR have been also associated with an increased risk of alcohol dependence.^97^ Interestingly, in this condition, a sex dimorphism of brain ALLO levels has been observed, with higher levels in the substantia nigra pars medialis of men.^98^

Mood disorders, in agreement with their sex dimorphism in term of incidence^99^, ^100^, ^101^, ^102^, ^103^, ^104^ and/or manifestations,^105^, ^106^, ^107^, ^108^, ^109^, ^110^, ^111^, ^112^, ^113^, ^114^, ^115^, ^116^, ^117^, ^118^, ^119^, ^120^, ^121^, ^122^, ^123^, ^124^, ^125^ may also alter the levels of ALLO in a sex‐dimorphic way. For example, the levels of this neuroactive steroid are decreased in the male, but not the female, brain mouse model of autism spectrum disorder‐like behaviour.^126^ In particular, in adult males, a decrease in the levels of this neuroactive steroid is associated with more severe restricted and repetitive behaviour.^127^

The plasma levels of ALLO are also decreased in association with post‐traumatic stress disorders (PTSD) re‐experiencing and depressive symptoms in PTSD patients, as well as with enhanced contextual fear memory and impaired fear extinction in PTSD experimental models.^128^, ^129^ Interestingly, in female PTSD patients, the observed low levels of ALLO in the CSF are associated with impairment of the enzyme synthesising this neuroactive steroid (ie, 3α‐HSOR).^130^ However, levels of ALLO are decreased in the medial orbital frontal cortex of male, but not female, PTSD patients.^131^

**FIGURE 2:**
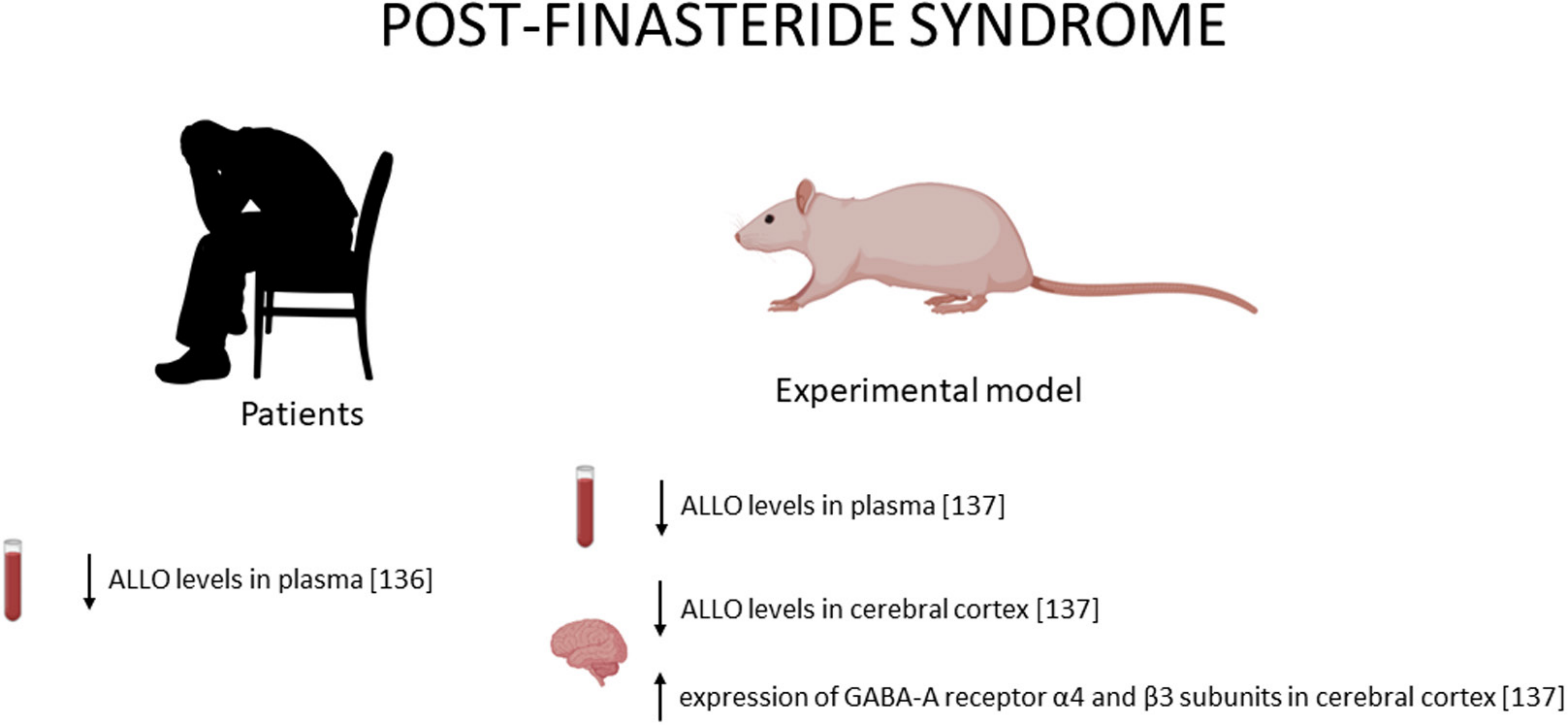
Allopregnanolone (ALLO) levels are decreased in plasma of PFS patients, as well as in its experimental model. In the male rat, the levels, as well as the GABA_A_ receptor composition, are also modified in the cerebral cortex. For details, see text. ALLO, allopregnanolone; GABA, γ‐aminobutyric acid; PFS, post‐finasteride syndrome

Another interesting example of alteration in ALLO levels is represented by post‐finasteride syndrome (PFS). Finasteride (commercially named Propecia or Proscar) is an inhibitor of two isoforms of the 5α‐R (ie, type 1 and 2), although it has higher affinity for type 2 in humans.^132^, ^133^ Approved in 1997 for the treatment of androgenetic alopecia at 1 mg day^‐1^, this drug has been shown to lead to a significant reduction in the progression of baldness and the stimulation of new hair growth.^134^ 5α‐R inhibitors have generally been described as well‐tolerated and relatively safe drugs; however, recent observations have led to a more critical re‐evaluation of these concepts (Figure 2). Indeed, 5α‐R inhibitors not only induced side effects during the treatment, but also they may persist after drug discontinuation inducing the so named PFS. Among these serious adverse side effects, there are sexual side effects (ie, low libido, erectile dysfunction, decreased arousal and difficulty in achieving orgasm), depression, anxiety and cognitive complaints.^135^ Data obtained in PFS patients show a decrease in the plasma levels of ALLO.^136^ It is interesting to note that, also in an experimental model of PFS, the plasma levels of this neuroactive steroid were decreased. This alteration was associated with a decrease in ALLO levels in the cerebral cortex,^137^ where a decrease in the gene expression of GABA_A_ receptor α4 and β3 subunits was observed^137^ (Figure 2).

#### 3.2.2 Neurodegenerative disorders

Altered levels of ALLO have been also reported in several neurodegenerative conditions and may differ in the two sexes, according to the sex‐dimorphic characteristics of neurodegenerative disorders.^104^, ^138^, ^139^, ^140^, ^141^, ^142^, ^143^, ^144^ For example, as reported in the caudate nucleus of Parkinson's disease (PD) patients, the 3α‐HSOR type 3 is up‐regulated.^145^ In the brain of an experimental model of PD (ie, mouse injected with 1‐methyl‐4‐phenyl‐1,2,3,6‐tetrahydropyridine), the levels of ALLO were increased in a manner similar to that occurring in the plasma of these animals.^146^ Accordingly, the block in ALLO production by the administration of a 5α‐R inhibitor, such as dutasteride, exerted protective effects on dopamine neurones in the same animal model^147^

Altered levels of ALLO are also detected in patients affected by multiple sclerosis (MS). For example, decreased levels of this neuroactive steroid have been detected in the the CSF of relapsing‐remitting MS male adult patients,^148^ as well as in brain samples of male MS patients.^149^ Observations in an experimental model of MS, such as the experimental autoimmune encephalomyelitis (EAE) rat MS model,^150^, ^151^ confirmed alterations in ALLO levels. Interestingly, these changes depend on the pathological phase considered, as well as the sex. For example, the levels of this neuroactive steroid increase at the acute phase of the disease (ie, 14 days post‐immunisation) in the spinal cord of males, but not females.^150^ By contrast, at the chronic phase (ie, 40 days post‐immunisation), no changes were reported in both sexes.^151^ The pattern in plasma is different. Indeed, at the acute phase, ALLO levels are decreased in females, but not males,^150^ whereas, at the chronic phase, the ALLO plasma levels are increased only in male animals.^151^ The levels of ALLO were altered in a sex‐dimorphic way also depending on the nervous region considered. Indeed, in the female, but not the male, cerebellum, ALLO levels are decreased both at the acute^150^ and chronic phase of the disease.^151^ In the male, but not the female, cerebral cortex, an increase in the levels of ALLO was observed at the acute phase of the disease.^150^ At the chronic phase, the levels of this neuroactive steroid were unaffected in the cerebral cortex of male and female rats.^151^ Sex differences in ALLO levels have been also detected in human relapsing‐remitting MS patients. Indeed, ALLO levels in the CSF are higher in male than in female patients.^152^ However, this difference is observed in the active, but not the stable, phase, where the levels are comparable in the two sexes.^152^

Brain levels of ALLO have been reported to be affected in a sex‐dimorphic way also in an experimental model of traumatic brain injury (TBI).^76^, ^153^, ^154^ Indeed, TBI decreased the brain levels of this neuroactive steroid in female mice,^153^ but not male mice.^154^

Diabetes mellitus alters central (ie, diabetic encephalopathy), as well as peripheral (ie, diabetic peripheral neuropathy), nervous function. ALLO levels are decreased in the cerebral cortex of both long‐term (ie, 3 months post‐induction) diabetic male and female rats.^155^ By contrast, the levels of this neuroactive steroid are decreased in the spinal cord of diabetic males, but not diabetic females.^155^ Long‐term diabetes also induced a decrease in ALLO levels in a peripheral nerve, such as the sciatic nerve, with altered levels in female animals, but not male animals.^155^ Similar to that reported in MS, and also in case of diabetes mellitus, alterations in the ALLO levels depend on the pathological phase considered. Indeed, short‐term diabetes (ie, 1 month postinduction) induces a decrease in the levels of this neuroactive steroid in the cerebral cortex and hippocampus of male animals.^156^, ^157^ In addition, an increase in the ALLO levels occurs only in the diabetic male sciatic nerve.^158^

Altered levels of this neuroactive steroid have been also reported in other animal models of peripheral neuropathy. For example, in the sciatic nerve of the sterol regulatory element binding protein‐1C knockout mice, a model of peripheral neuropathy as a result of the ablation of the key lipogenic transcription factor,^159^ the levels of ALLO are increased at 10 months of age compared to those observed in wild‐type animals.^160^ The crush injury of the rat sciatic nerve induced a decrease in the levels of ALLO, in agreement with the reduced levels of its precursor, DHP. These events may be associated with a decrease in the expression of enzyme 5α‐R in the distal portion of the injured nerve.^161^

An important component of the peripheral neuropathy is the neuropathic pain. As demonstrated in an animal model of neuropathic pain induced by peripheral nerve injury, the levels of ALLO are increased in the spinal cord, together with increased expression and activity of 3α‐HSOR.^162^ As proposed, the increase in the levels of neuroactive steroid and its synthesising enzyme, 3α‐HSOR, appears to be an adaptive response to cope with pain.^163^, ^164^ Indeed, an increase in ALLO levels has been reported in the rat lateral thalamus (ie, an important brain region for pain modulation) after spared nerve injury.^165^

## 4 EFFECTS OF ALLO UNDER PHYSIOLOGICAL AND PATHOLOGICAL CONDITIONS

### 4.1 Physiological effects

ALLO regulates lordosis and other motivated behaviours^166^ by its action on GABA_A_ receptors located in the midbrain ventral tegmental area.^167^, ^168^ However, this action appears to be mediated not only by this neurotransmitter receptor, but also by PROG receptor because the administration of mifepristone (ie, an antagonist of PROG receptor) inhibits the induction of this behavioural response to ALLO.^169^, ^170^ This effect can be explained based on the ability of ALLO to be retro‐converted into DHP by 3α‐HSOR.^171^, ^172^, ^173^

A critical role for ALLO has been also demonstrated in brain maturation. The physiological fluctuations of this steroid occurring during rodent fetal life and after birth^80^ may contribute to maintaining the low level of arousal activity, characteristic of fetal brain.^174^ In addition, neonatal levels of ALLO promote the formation of neuronal circuitry and support the survival of developing neurones.^175^ Moreover, this neuroactive steroid is involved in the structural formation of the cerebral cortex, thalamus and hippocampus.^176^, ^177^ Furthermore, ALLO is involved in myelin formation of the CNS.^12^ However, this neuroactive steroid is not only important for brain fetal maturation, but also for the pregnant mother. Indeed, during pregnancy, an increase of ALLO levels occurs in the maternal peripheral circulation, as well as in the maternal brain.^178^, ^179^ In rats, the increased levels of this neuroactive steroid interfere with the hypothalamic‐pituitary‐adrenal (HPA) axis reducing, in particular during late pregnancy, the response to stress exposure of the mother.^180^, ^181^, ^182^, ^183^

ALLO exerts a crucial role also in the adult brain. At this stage, the enzymatic complex 5α‐R/3α‐HSOR co‐localises in glutamatergic and GABAergic neurones of the cerebral cortex, hippocampus, amygdala and thalamus, suggesting that its activity is relevant for the synthesis and the effects of neurotransmitters in these cells.^34^ Indeed, ALLO is able to increase the protein content of glutamic acid decarboxylase in the olfactory bulb.^184^ In addition, this steroid regulates the neuronal cytoskeleton because its administration to ovariectomised animals decreases microtubule‐associated protein Tau and glycogen synthase kinase 3β expression in the cerebellum.^185^

ALLO is also involved in the mood regulation. For example, together with glucocorticoids, this neuroactive steroid regulates the stress response. Thus, an increase in the ALLO levels has been reported in plasma and cerebral cortex of adult male rats after swim stress.^186^

ALLO is also able to regulate the dopaminergic system.^187^, ^188^, ^189^, ^190^ In an experimental model in which dopaminergic signalling was altered (ie, animals reared in social isolation), a decrease in the levels of ALLO occurred in the brain but not in plasma.^191^ In addition, in the foot shock stress model, treatment with this neuroactive steroid stimulates the extracellular dopamine release from cortical dopaminergic neurones,^192^ and prevents the dopamine increase in the cerebral cortex and in the nucleus accumbens.^193^ Moreover, ALLO modulates the levels and metabolism of this neurotransmitter during the oestrous phase of the female ovarian cycle. Indeed, it decreases the levels of dopamine and the dopamine metabolite 3,4‐dihydroxyphenylacetic acid in the striatum,^194^ as well as the dopamine output in the nucleus accumbens and prefrontal cortex in freely moving rats.^193^ In addition, females showing high progesterone levels (ie, in pro‐oestrus) are less responsive to ALLO treatment than in other oestrous phases.^195^

Interestingly, it has been proposed that ALLO may also affect the enzymatic activity of the DNA base excision repair (BER) pathway. Indeed, as recently reported in both natural and stressful conditions, the treatment with this neuroactive steroid is able to modulate the synthesis of BER pathway enzymes in sheep hippocampus and amygdala.^196^

Physiological effects of ALLO have been also reported in the PNS. In Schwann cells, ALLO treatment enhances GABA synthesis through an increased expression of glutamic acid decarboxylase^197^ and also promotes glutamate uptake through an increase in the excitatory amino acid carrier 1.^198^ ALLO treatment is also able to regulate, in peripheral nerves and Schwann cells, the expression of specific transcription factors involved in the myelination process (ie, Krox‐20)^199^ and the expression of a myelin protein (ie, peripheral myelin protein 22, PMP22).^200^, ^201^ An antagonist of the GABA_A_ receptor, such as the bicuculline, is able to completely abolish the stimulatory effect exerted by ALLO on PMP22 in Schwann cell cultures. In addition, a GABA_A_ receptor agonist (ie, muscimol) shows a stimulatory effect on PMP22 that was comparable to that of ALLO.^202^ These observations, together with the finding that peripheral nerves, as well as Schwann cells, express GABA_A_ receptors,^15^, ^200^ may suggest that the effect on peripheral myelin are mediated by the GABA_A_ receptor.^200^, ^201^ Indeed, isoallopregnanolone, which does not directly interact with GABA_A_ receptor, does not alter PMP22 expression. Interestingly, the effect of ALLO on the expression of myelin proteins is sex‐dimorphic. Indeed, the treatment with this neuroactive steroid increases the expression of PMP22 and of another myelin protein, such as glycoprotein zero, in female rat Schwann cells, but not in male cells.^203^

### 4.2 Effects of ALLO in pathological conditions

**FIGURE 3:**
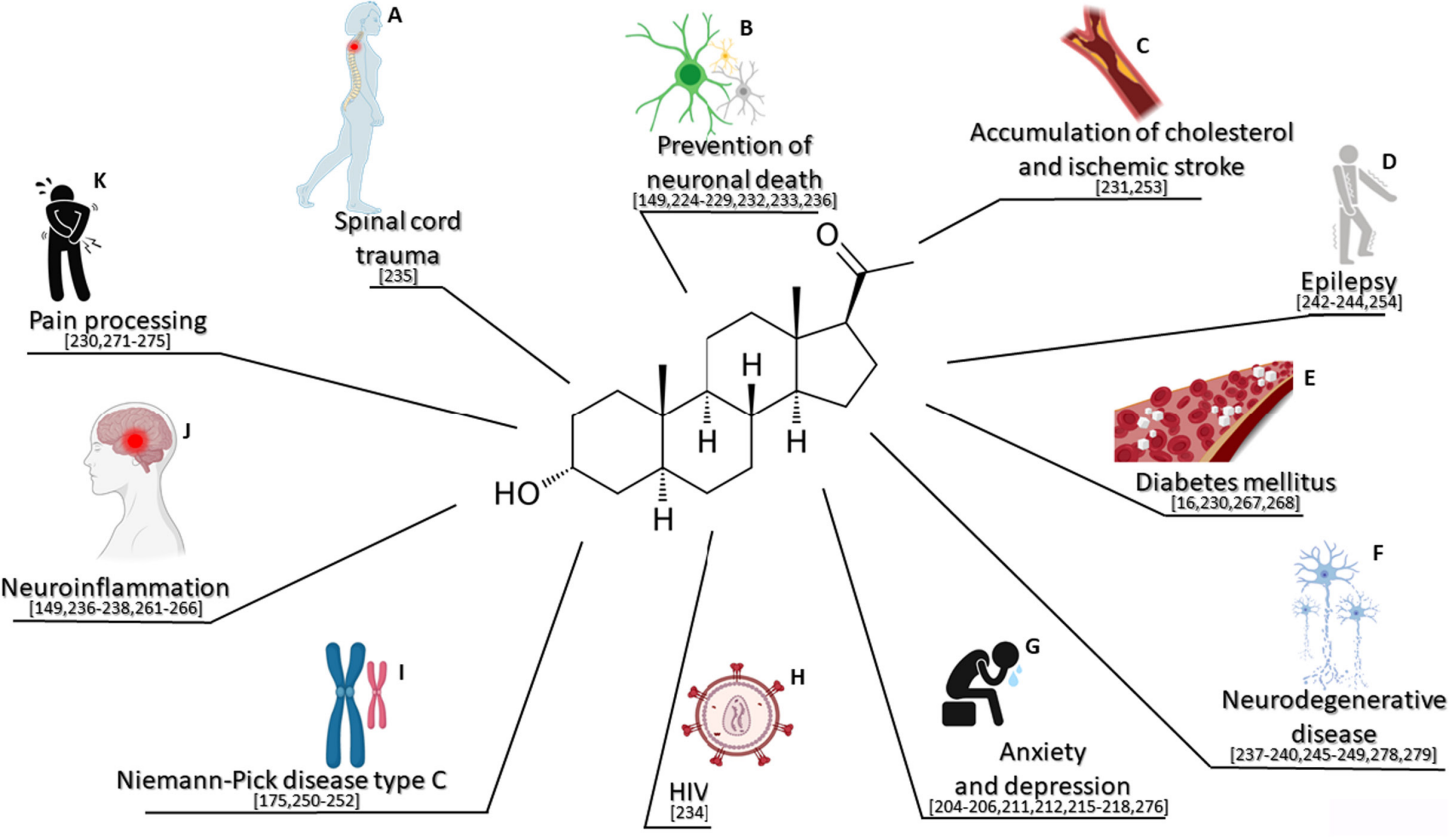
Neuroprotective effects of allopregnanolone. Treatment with this neuroactive steroid shows: (A) beneficial effects on spinal cord trauma, (B) prevention of neuronal death, (C) reduction of cholesterol accumulation and stroke, (D) decrease in epileptic events, (E) beneficial effects in nervous damage induced by diabetes mellitus, (F) protective effects on neurodegenerative diseases (eg, Alzheimer’s disease, Parkinson's disease and amyotrophic lateral sclerosis), (G) anxiolytic and anti‐stress actions, (H) effects against the neurotoxicity exerted by human immunodeficiency virus (HIV), (I) protective effects in an experimental model of Niemann‐Pick type C and in (J) neuroinflammatory conditions (eg, multiple sclerosis and experimental autoimmune encephalomyelitis), and (K) analgesic effects against neuropathic pain

The therapeutic potential of ALLO has been explored in different pathological conditions, demonstrating interesting beneficial effects (Figure 3). ALLO treatment exerts anxiolytic and anti‐stress actions.^204^, ^205^ Activation of GABA_A_ receptors by this neuroactive steroid appears to be responsible for these effects.^206^ Interestingly, corticotrophin‐releasing hormone (CRH) neurones, the primary regulators of the HPA axis, are regulated by GABAergic inhibition.^207^ In particular, it has been shown that CRH neurones are controlled by delta (δ)‐containing GABA_A_ receptors.^208^ In agreement, in vitro studies showed that the human CRH promoter activity was inhibited by ALLO after basal or forskolin‐induced promoter activity.^209^ In addition, in virgin female rats, ALLO administration was able to reduce CRH gene expression in the parvocellular paraventricular nucleus.^180^ Similarly, recent evidence in sheep has demonstrated that, in stressful conditions, this neuroactive steroid reduced CRH gene expression, as well as pro‐opiomelanocortin expression, in anterior pituitary, resulting in diminished levels of plasma adrenocorticotrophic hormone and cortisol.^210^ By contrast, the anti‐depressive effect exerted by ALLO, at least in the forced swimming model, appears also to involve the stimulation of dopamine D2‐like receptors.^211^ In addition, it has been observed that, in the nucleus accumbens of learned helplessness rats (ie, an experimental model of depression), the astroglial glutamate transporter‐1 and glutamine synthetase system is normalised by ALLO treatment.^212^ In this context, it is interesting to note that effective antidepressant treatment is able to increase the reduced levels of ALLO observed in depressed patients.^213^ In agreement, in an experimental model, the antidepressant fluoxetine was able to increase ALLO levels.^214^ Interestingly, in mood and anxiety disorders, ALLO treatment shows sex‐specific features. Indeed, this neuroactive steroid attenuates in females, but not in males, the HPA axis responses to interleukin‐1β in adult prenatally stressed rats.^215^ Also only in females, ALLO treatment blocks the stress‐induced reinstatement of cocaine‐seeking behaviour induced by yohimbine.^216^ ALLO treatment before stress reduced basal CRF mRNA expression in male rats.^217^ Interestingly, recent observations obtained in rats show sex‐ and brain region‐specific regulation of CRF after ALLO treatment, suggesting new sex‐specific therapeutic approaches based on this neuroactive steroid for stress‐related disorders and addiction.^218^

Despite ALLO treatment shows anxiolytic effects, women with premenstrual dysphoric disorder show an altered sensitivity to this neuroactive steroid over the menstrual cycle compared to healthy controls.^219^ In these patients, the negative mood symptoms are antagonised by isoallopregnanolone treatment in the premenstrual phase, reducing negative mood symptoms in premenstrual dysphoric disorder.^220^ As suggested, a possible hypothesis for this paradoxical effect could be changes in GABA_A_ receptor composition (ie, an up‐regulation of the α4, β, δ subunit expression) during the luteal phase.^221^

Similarly, in D1CT‐7 mice (ie, an experimental model of Tourette syndrome), ALLO treatment exacerbated the Tourette syndrome symptoms,^222^ whereas isoallopregnanolone administration is able to reduce the number of tic‐like behaviours induced by stress.^223^

ALLO treatment has also been reported to exert protective effects in experimental models of neurodegeneration. For example, this neuroactive steroid is protective against kainic acid‐induced excitotoxicity in the hippocampus in vivo,^224^ reduces seizures,^225^, ^226^, ^227^, ^228^, ^229^ prevents cell apoptosis in the spinal cord of streptozotocin (STZ) diabetic rats,^230^ and protects against stroke,^231^ oxygen‐glucose deprivation,^232^ TBI^233^ and the neurotoxic effects exerted by human immunodeficiency virus.^234^

ALLO treatment exerts protective effect also in spinal cord trauma. For example, in organotypic spinal cord cultures put under injury (ie, a weight drop model), this neuroactive steroid, by activation of GABA_A_ receptors, is able to decrease membrane damage and prevent neuronal death.^235^

ALLO is also effective in experimental model of MS, such as EAE, where the treatment reduces axonal injury,^149^, ^236^ as well as in Alzheimer’s disease (AD) models, where it is able to induce neurogenesis/oligodendrogenesis and to reduce β‐amyloid levels^237^, ^238^ and bioenergetics deficits.^239^ In particular, for the neuroprotective effects of i.v. ALLO treatment in AD, the dosing and treatment regimen appears to be crucial.^237^, ^238^, ^240^ By contrast, intranasal delivery of this neuroactive steroid has been proposed as an excellent therapeutic strategy against seizures.^241^ In this context, it is important to highlight that neuroactive steroids represent an important target for the treatment of focal epileptic disorders.^242^ Indeed, alteration of ALLO synthesis modulate status epilepticus dynamics.^243^, ^244^ In addition, protective effects have been reported in an experimental model of amyotrophic lateral sclerosis (ie, Wobbler mouse),^245^ in PD experimental models,^246^, ^247^ as well as in a pilot clinical study performed in patients affected by fragile X‐associated tremor/ataxia syndrome, where the ALLO treatment was reported to improve cognitive function and neurodegeneration.^248^, ^249^

In an animal model of Niemann‐Pick type C disease, this neuroactive steroid has been demonstrated to delay the onset of neurological symptoms, increasing Purkinje and granule cell survival in the cerebellum, reducing cortical ganglioside accumulation, cholesterol accumulation and inflammation, and enhancing myelination.^175^, ^250^, ^251^ Interestingly, the combination of this neuroactive steroid with cyclodextrin and miglustat seems to ameliorate motor but not cognitive deficits.^252^

Few experimental observations have been performed to evaluate possible sex difference in the protective effects of ALLO on neurodegeneration. As demonstrated, a low dose of this neuroactive steroid induces a higher neuroprotection from ischaemic damage in females compared to males.^253^ In an animal model of epilepsy, the treatment shows greater antiseizure potency in females than in males; this effect was associated with higher levels of extrasynaptic delta subunit of GABA_A_ receptors in female animals.^254^

An important aspect in neurodegenerative and psychiatric diseases is the neuroinflammation.^255^, ^256^, ^257^, ^258^, ^259^, ^260^ Indeed, ALLO exerts a variety of protective effects in this process. For example, this neuroactive steroid reduces protein‐protein interactions initiating toll‐like receptor 4 (TLR4)‐dependent signalling in immune cells and the brain^261^ alongside of TLR7.^262^ In addition, its treatment decreases microglia reactivity and lymphocyte infiltration in an EAE experimental model,^149^, ^236^ as well as neuroinflammatory burden in AD models.^237^, ^238^ A protective effect has been also reported in ischaemic stroke, where its treatment down‐regulates the production of pro‐inflammatory cytokines (ie, tumour necrosis factor‐α and interleukin‐6) protecting against blood‐brain barrier disruption and reducing infarct size.^263^ Finally, after TBI, ALLO decreases the expression levels of interleukin‐1β and tumour necrosis factor‐α, in the rat brain,^264^ and increases a potent inhibitor of the complement convertases that are activators of the inflammatory cascade.^265^ Indeed, it has been recently demonstrated that administration of this neuroactive steroid to primary cell cultures or to a microglial cell line (ie, BV‐2), induces changes in morphology and phagocytic activity in microglial cells.^266^ These results might help to shed light on the protective mechanisms of ALLO in inflammatory conditions. Protective effects of ALLO have been also reported in peripheral neuropathies. For example, in an experimental model of peripheral diabetic neuropathy (ie, rats rendered diabetic by streptozotocin injection), ALLO treatment improves nerve conduction velocity, thermal threshold, mRNA levels of a myelin protein such as PMP22, and skin innervation density.^267^ In addition, this neuroactive steroid is also able to counteract myelin abnormalities in rat peripheral nerves induced by the ageing process.^16^, ^268^

Neuropathic pain is another important component of the damage in the PNS and CNS. In this context, it is important to note that 3α‐HSOR is expressed in pain information processing areas of the CNS, such as the dorsal root ganglia and the dorsal horn of the spinal cord.^163^, ^269^ Indeed, blockade of 3α‐HSOR and the consequent inhibition of the local synthesis of THP in these two compartments enhances neuropathic pain induced by sciatic nerve injury.^163^, ^164^ In addition, the synthesis of ALLO in the dorsal horn of the spinal cord is regulated by an important neuropeptide involved in pain processing, such as substance P.^270^ Altogether, these observations suggest that endogenous ALLO is involved in pain processing. From this point of view, ALLO exerts analgesic effects. For example, treatment with this neuroactive steroid ameliorates diabetic‐induced thermal hyperalgesia in the STZ model.^230^ In addition, it suppresses allodynia/hyperalgesia evoked by antineoplastic drugs, such as vincristine^271^ or oxaliplatin,^272^ or by spinal nerve ligation.^273^ The analgesic actions of ALLO appear to be mediated by the potentiation of GABA_A_ receptor activity and the inhibition of T‐type Ca^2+^ channels.^274^, ^275^

**FIGURE 4:**
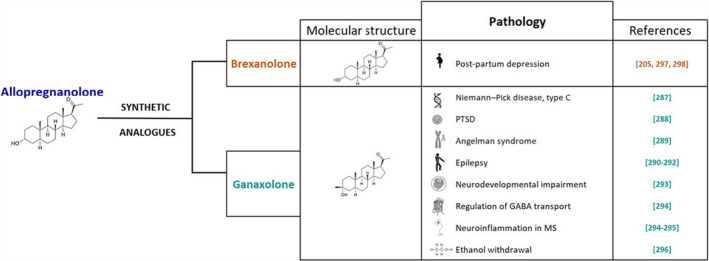
Protective effects exerted by allopregnanolone analogues, brexanolone and ganaxolone, in different neuropathologies. For details see text. PTSD, post‐traumatic stress disorder; MS, multiple sclerosis

Altogether, these observations indicate that ALLO may be considered as a potential candidate for the treatment of psychiatric,^276^ traumatic^277^ and neurodegenerative disorders.^278^, ^279^ However, one of the disadvantages of the treatment with natural ALLO is represented by its rapid metabolism and their low oral bioavailability.^87^ On this basis, extensive research has been devoted to synthesising analogues of ALLO,^280^, ^281^, ^282^, ^283^ showing promising neuroprotective effects.^205^, ^277^, ^284^, ^285^, ^286^ In particular, as depicted in Figure 4, two synthetic analogues, such as ganaxolone and brexanolone, appear to be very promising. Indeed, ganaxolone has been demonstrated to be neuroprotective in an experimental model of Niemann‐Pick type C,^287^ in an animal model of PTSD,^288^ in Angelman syndrome,^289^ and in animal models of epilepsy and related conditions.^290^, ^291^, ^292^ In addition, its treatment is able to reduce neurodevelopmental impairment following preterm birth,^293^ to regulate GABA transport and neuroinflammation in MS,^294^ to induce remyelination in focal demyelination of the corpus callosum ^295^ and to be effective for the treatment of ethanol withdrawal‐induced seizures.^296^

Brexanolone has been recently approved by the US Food and Drug Administration for the specific treatment of post‐partum depression,^205^, ^297^, ^298^ even some concerns regarding its use have been also raised^299^, ^300^ (Figure 4).

An alternative to the use of synthetic steroids is to stimulate the endogenous synthesis of ALLO. One option is the activation of steroidogenesis with ligands of TSPO, a part of the macromolecular complex involved in the transfer of cholesterol into mitochondria (ie, the first step of the steroidogenesis).^301^ Indeed, treatment with TSPO ligands has been reported to exert neuroprotective effects, such as in EAE mice using etifoxine^302^ or XBD173,^303^ in rat models of PTSD administered with midazolam^304^ or YL‐IPA08,^305^ in a rat ex vivo glaucoma model with PK11195^306^ and in diabetic rats with Ro5‐4864 (^307^, ^308^) or AC‐5216.^309^

Another possibility is the activation of liver X receptors (LXRs). Indeed, treatment with a LXR ligand such as the GW3965 increases the levels of ALLO in the spinal cord and the cerebral cortex, as well as the levels of its precursor, DHP, in the sciatic nerve of diabetic rats.^307^, ^310^ This, in turn, exerts neuroprotective effects on thermal nociceptive activity, nerve conduction velocity and Na^+^,K^+^‐ATPase activity.^310^

## 5 CONCLUSIONS

As defined many decades ago,^311^ neuroactive steroids represent important physiological modulators of the nervous system. They are involved in basic processes such as myelination, neuronal transmission and brain maturation. Among the natural neuroactive steroids, ALLO has received particular attention because of its relevance in such processes. Concerning ALLO physiology, many issues have to be taken into account. For example, its levels are linked to the expression of the enzymatic complex of 5α‐R/3α‐HSOR, thus producing a different profile in relation to the nervous structure being considered. In addition, neuroactive steroid plasma levels, as well as the sex, have an influence on the levels of ALLO in the nervous system.

In addition, as more recently explored, the neuroactive steroids are also neuroprotective agents. Among them, ALLO appears to be particularly relevant because of its implication in neuropathological situations. Up to now, its importance in depression and anxiety, in neurodegenerative diseases (eg, AD, PD and diabetes mellitus), in traumatic events (eg, spinal cord trauma, nerve injury), and in inflammatory environments (eg, MS, ischaemia), is becoming increasingly evident. ALLO exerts its protective effects mainly by interaction with the GABA_A_ receptor, although, as a result of the ability of the enzyme 3α‐HSOR to retro‐convert ALLO into DHP, this steroid may also interact with PROG receptor. The unfavourable pharmacokinetic of ALLO limits its therapeutic potential, as observed in many experimental paradigms. Thus, alternative strategies have been explored. For example, synthetic analogues have been successfully applied to several pathological conditions, also leading to its inclusion in clinical practice. An alternative to the synthetic ALLO derivative administration is represented by the pharmacological stimulation of steroidogenesis, and consequently ALLO synthesis, by specific ligands.

In conclusion, a deeper investigation of the mechanisms involved in the protective effects of neuroactive steroids in general, and of ALLO in particular, is needed to propose new therapeutic strategies based on this neuroactive steroid for the treatment of neuropathological conditions.

## AUTHOR CONTRIBUTIONS

**Silvia Diviccaro:** Conceptualisation; Writing – original draft; Writing – review & editing. **Lucia Cioffi:** Writing – review & editing. **Eva Falvo:** Visualisation. **Silvia Giatti:** Conceptualisation; Writing‐original draft; Writing – review & editing. **Roberto Cosimo Melcangi:** Conceptualisation; Writing‐original draft; Writing – review & editing.

### PEER REVIEW

The peer review history for this article is available at https://publons.com/publon/10.1111/jne.12996.
